# Supplementary material for: Population genomics identifies a distinct Plasmodium vivax population on the China-Myanmar border of Southeast Asia
Source: PLoS Negl Trop Dis. 2020 Aug 3;14(8):e0008506. doi: 10.1371/journal.pntd.0008506 (PMC7425983; doi:10.1371/journal.pntd.0008506)
Supplement: S1 Table — (PDF) [file pntd.0008506.s001.pdf]

Supplemental Table 1. DNA processing results for 23 samples from the China-Myanmar border

	DNA input (ng)	Read Pairs	Surviving Read Pairs	% Read Survival	Average Depth
**LZCH1337**	100	8,387,821	623,995	7.4%	4.54
**LZCH1403**	100	8,187,840	404,309	4.9%	2.25
**LZCH1436**	100	8,270,987	1,398,859	16.9%	11.72
**LZCH1447**	69	8,198,480	6,134,476	74.8%	48.90
**LZCH1474**	70	7,252,842	6,663,732	91.9%	61.70
**LZCH1476**	66	7,855,284	6,276,970	79.9%	58.04
**LZCH1510**	100	7,939,046	1,115,736	14.1%	9.14
**LZCH1592**	100	7,421,681	3,601,708	48.5%	33.01
**LZCH1596**	100	8,681,908	872,021	10.0%	6.97
**LZCH1597**	100	8,153,287	4,786,037	58.7%	44.31
**LZCH1599**	100	8,931,072	3,278,840	36.7%	29.13
**LZCH1708**	58	9,197,595	3,962,420	43.1%	36.72
**LZCH1720**	55	8,522,156	6,464,904	75.9%	60.08
**LZCH1751**	100	9,249,215	1,728,877	18.7%	15.15
**LZCH1779**	100	8,735,962	1,287,803	14.7%	10.86
**LZCH1809**	100	8,443,602	978,208	11.6%	8.00
**LZCH1824**	50	8,045,163	1,801,450	22.4%	15.89
**LZCH1873**	100	9,004,474	670,303	7.4%	4.93
**LZCH1886**	65	8,891,165	6,399,607	72.0%	59.23
**NB43**	100	7,436,921	5,094,511	68.5%	47.06
**NB45**	100	7,671,822	6,344,125	82.7%	58.51
**NB48**	100	9,529,810	3,783,355	39.7%	34.59
**NB59**	100	8,759,135	376,875	4.3%	2.16
	**Median**	8,387,821	3,278,840	36.7%	29.13
