# Supplementary material for: Population genomics identifies a distinct Plasmodium vivax population on the China-Myanmar border of Southeast Asia
Source: PLoS Negl Trop Dis. 2020 Aug 3;14(8):e0008506. doi: 10.1371/journal.pntd.0008506 (PMC7425983; doi:10.1371/journal.pntd.0008506)
Supplement: S3 Table — Italicized regions on the right were only removed during selection scans, where excessive loss of polymorphism from removal of high-CV SNPs may result in selection bias. (PDF) [file pntd.0008506.s003.pdf]

Supplemental Table 3. Regions removed due to high chances of mismapping.

Chrom	Start	End	Explanation	*Chrom*	*Start*	*End*	*Explanation*
PvP01_12_v1	792292	818496	MSP7	*PvP01_01_v1*	*0*	*102166*	*Subter1*
PvP01_10_v1	1330765	1364791	MSP3	*PvP01_01_v1*	*896664*	*1021664*	*Subter2*
PvP01_01_v1	0	7914	Subter1	*PvP01_02_v1*	*0*	*95632*	*Subter1*
PvP01_01_v1	936372	1021664	Subter2	*PvP01_02_v1*	*706327*	*956327*	*Subter2*
PvP01_02_v1	0	145	Subter1	*PvP01_03_v1*	*0*	*100000*	*Subter1*
PvP01_02_v1	809467	956327	Subter2	*PvP01_03_v1*	*807034*	*896704*	*Subter2*
PvP01_03_v1	0	2435	Subter1	*PvP01_04_v1*	*0*	*200000*	*Subter1*
PvP01_04_v1	0	11544	Subter1	*PvP01_04_v1*	*910822*	*1012024*	*Subter2*
PvP01_04_v1	1001829	1012024	Subter2	*PvP01_05_v1*	*0*	*152481*	*Subter1*
PvP01_05_v1	0	4121	Subter1	*PvP01_05_v1*	*1372333*	*1524814*	*Subter2*
PvP01_05_v1	1508319	1524814	Subter2	*PvP01_06_v1*	*0*	*104279*	*Subter1*
PvP01_06_v1	1029030	1042791	Subter2	*PvP01_06_v1*	*938512*	*1042791*	*Subter2*
PvP01_07_v1	0	7803	Subter1	*PvP01_07_v1*	*0*	*165221*	*Subter1*
PvP01_07_v1	1474452	1652210	Subter2	*PvP01_07_v1*	*1486989*	*1652210*	*Subter2*
PvP01_08_v1	1648611	1761288	Subter2	*PvP01_08_v1*	*0*	*176128*	*Subter1*
PvP01_09_v1	0	183564	Subter1	*PvP01_08_v1*	*1585160*	*1761288*	*Subter2*
PvP01_09_v1	2235275	2237066	Subter2	*PvP01_09_v1*	*0*	*250000*	*Subter1*
PvP01_10_v1	0	5329	Subter1	*PvP01_09_v1*	*2013360*	*2237066*	*Subter2*
PvP01_10_v1	1517016	1548844	Subter2	*PvP01_10_v1*	*0*	*154884*	*Subter1*
PvP01_11_v1	2056852	2131221	Subter2	*PvP01_10_v1*	*1393960*	*1548844*	*Subter2*
PvP01_12_v1	0	5873	Subter1	*PvP01_11_v1*	*0*	*213122*	*Subter1*
PvP01_12_v1	3085629	3182763	Subter2	*PvP01_11_v1*	*1918099*	*2131221*	*Subter2*
PvP01_13_v1	0	23338	Subter1	*PvP01_12_v1*	*0*	*318276*	*Subter1*
PvP01_13_v1	1898648	2093556	Subter2	*PvP01_12_v1*	*2864487*	*3182763*	*Subter2*
PvP01_14_v1	3108714	3153402	Subter2	*PvP01_13_v1*	*0*	*209355*	*Subter1*
				PvP01_13_v1	*1884201*	*2093556*	*Subter2*
				PvP01_14_v1	*0*	*315340*	*Subter1*
				PvP01_14_v1	*2838062*	*3153402*	*Subter2*

Italicized regions on the right were only removed during selection scans, where excessive loss of polymorphism from removal of high CV SNPs may result in selection bias.
