# Supplementary material for: Population genomics identifies a distinct Plasmodium vivax population on the China-Myanmar border of Southeast Asia
Source: PLoS Negl Trop Dis. 2020 Aug 3;14(8):e0008506. doi: 10.1371/journal.pntd.0008506 (PMC7425983; doi:10.1371/journal.pntd.0008506)
Supplement: S4 Table — (PDF) [file pntd.0008506.s004.pdf]

Supplemental Table 4. Fst values corrected for structure by condensing of IBD clusters.

	CMB	Thailand	Cambodia
**Thailand**	0.0190 (*0.0186, 0.0195*)		
**Cambodia**	0.0296 *(0.0290, 0.0301)*	0.0137 *(0.0136, 0.0138)*	
**Vietnam**	0.0309 *(0.0303, 0.0316)*	0.0007 *(0.0003, 0.0011)*	-0.0095 *(-0.0098, -0.0091)*
