# Supplementary figures and images for: Population genomics identifies a distinct Plasmodium vivax population on the China-Myanmar border of Southeast Asia
Source: PLoS Negl Trop Dis. 2020 Aug 3;14(8):e0008506. doi: 10.1371/journal.pntd.0008506 (PMC7425983; doi:10.1371/journal.pntd.0008506)

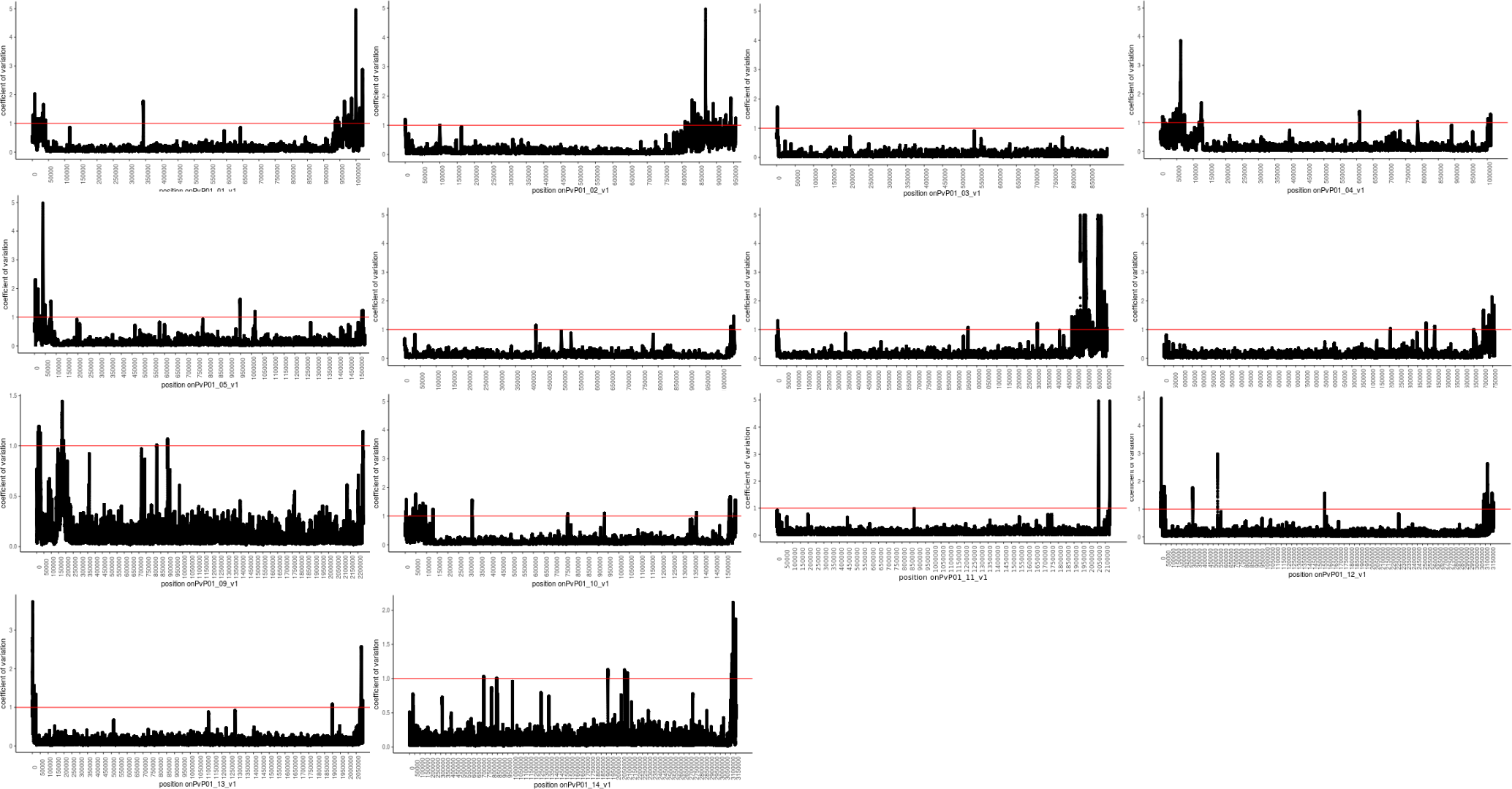

Supplement: S1 Fig — CV = 1 is marked in red to show cutoff for pruning due to high likelihood of mismapping. For chromosomes where CV in some case rose above 5, values above 5 were excluded from plotting, but were still masked. (TIF) [file pntd.0008506.s005.tif]

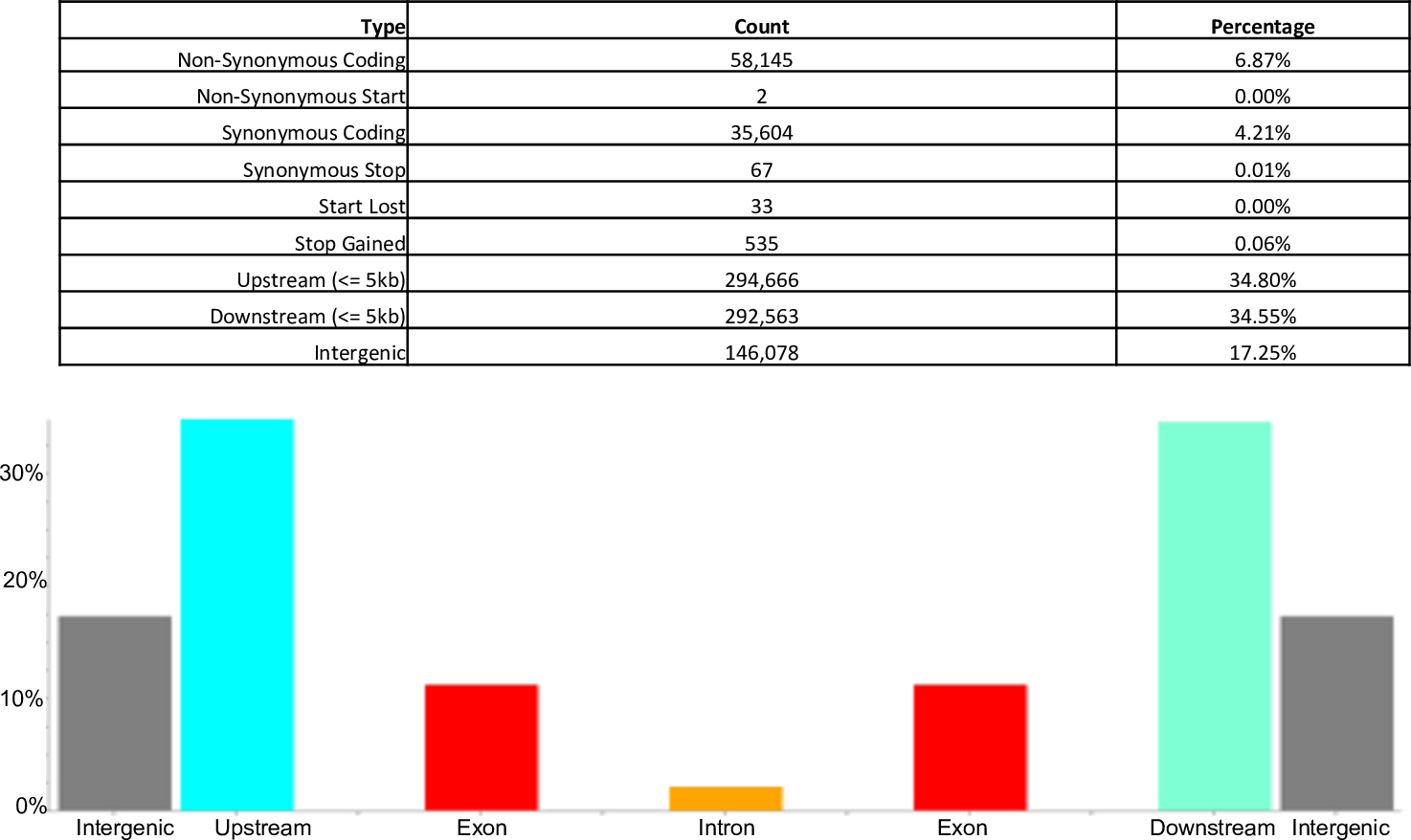

Supplement: S2 Fig — Potential effects from each variant are categorized into various functional (top panel) and regional (bottom panel) categories. Variants may have more than one effect. (TIF) [file pntd.0008506.s006.tif]

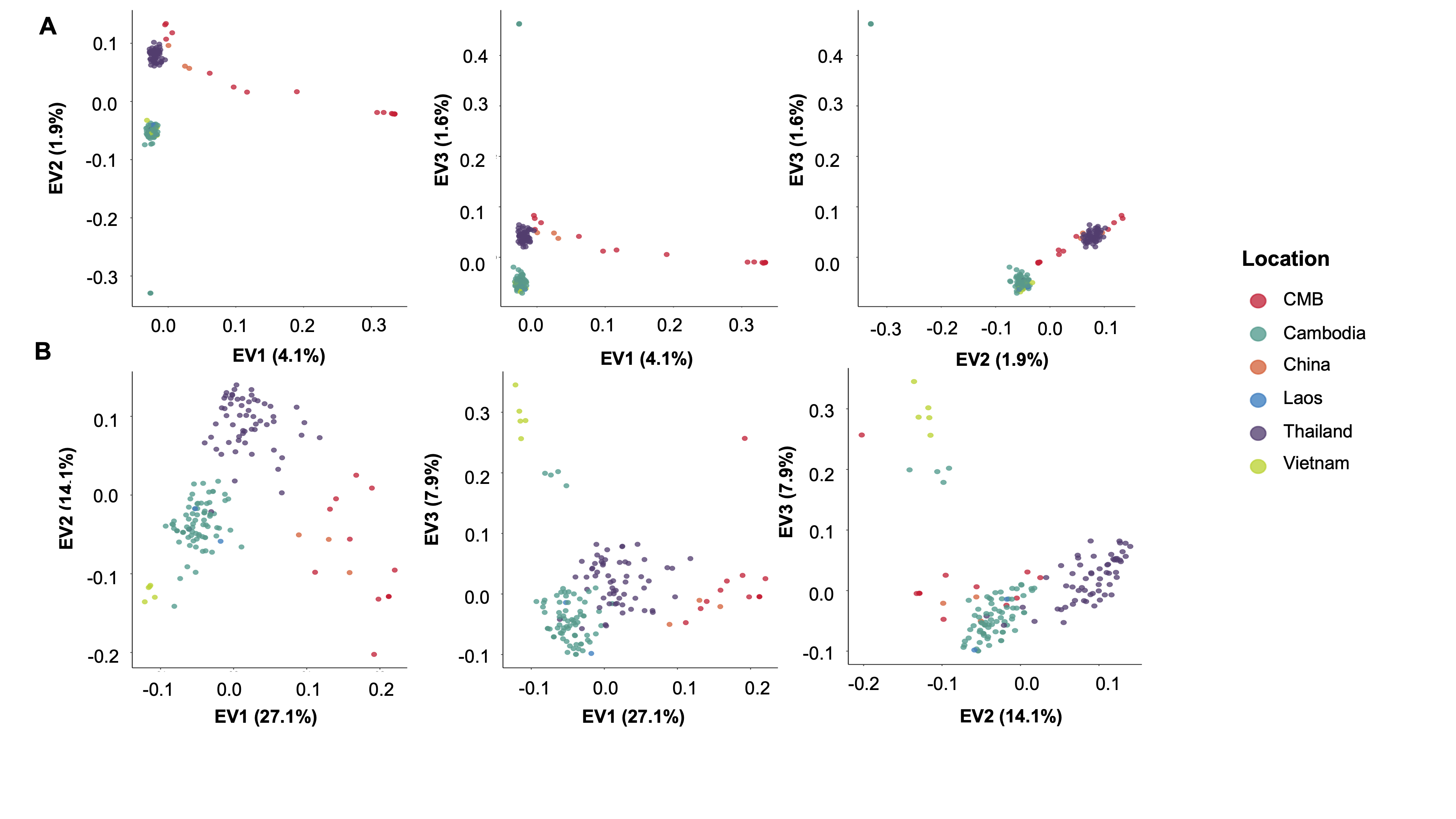

Supplement: S3 Fig — A) All SNPs, B) High-FST barcode only. (TIFF) [file pntd.0008506.s007.tiff]

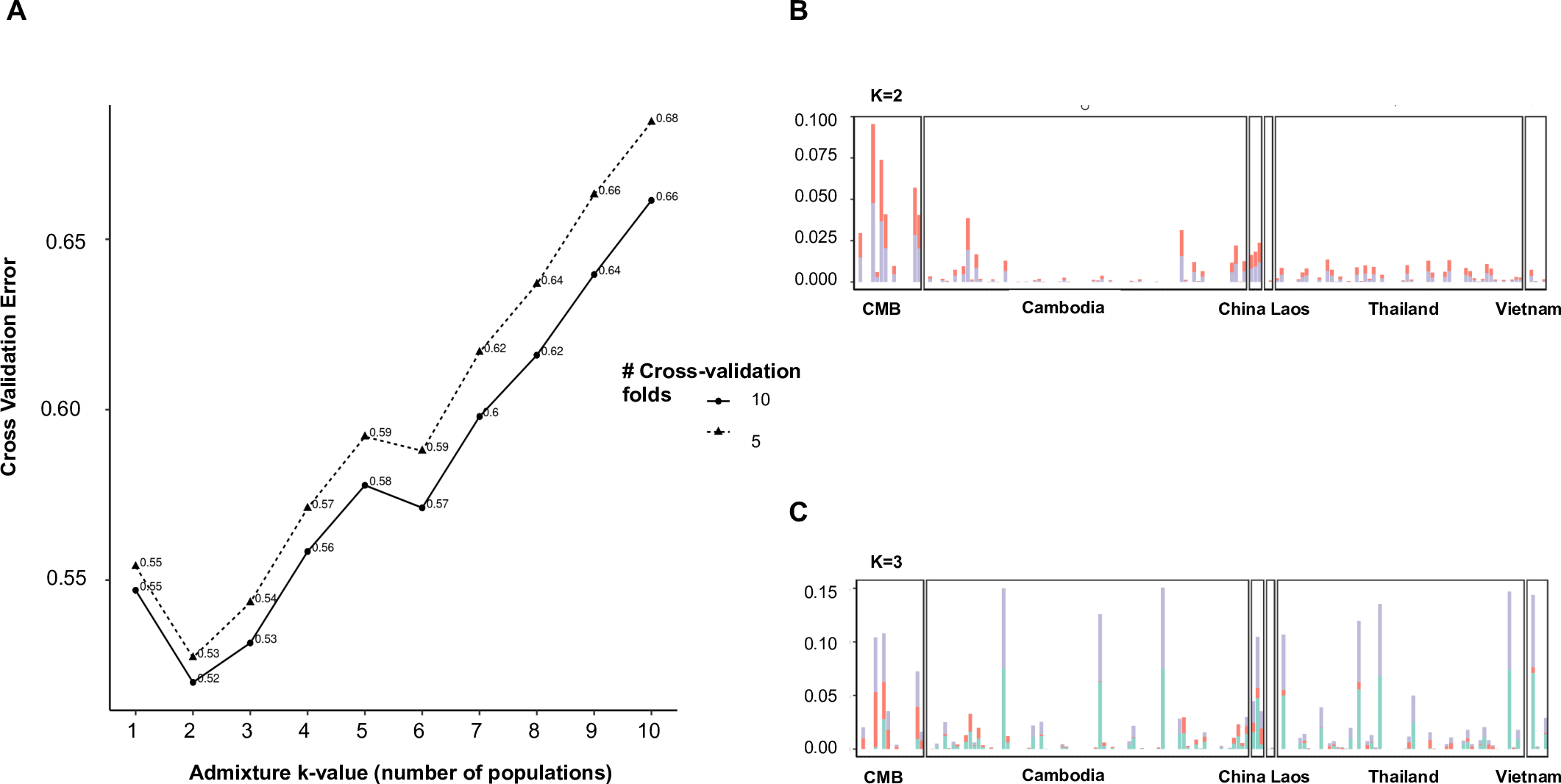

Supplement: S4 Fig — A) K-value cross validation scores for admixture analysis at 5 and 10 folds. B&C) Standard error across 1000 bootstraps for (B) k = 2 and (C) k = 3 admixture models. (TIF) [file pntd.0008506.s008.tif]

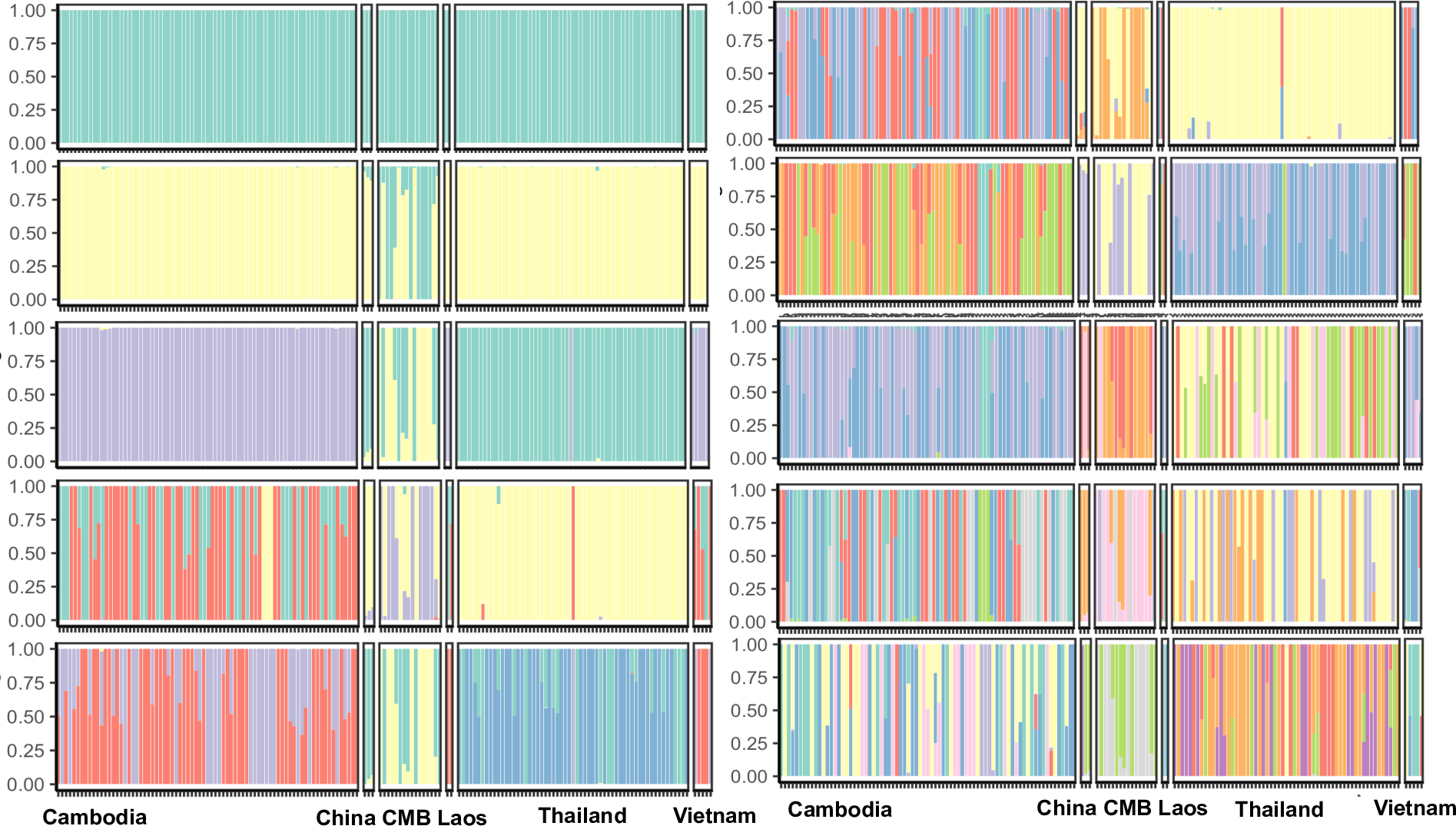

Supplement: S5 Fig — K value gets ascendingly larger going from top to bottom on the left than the right column. (TIF) [file pntd.0008506.s009.tif]

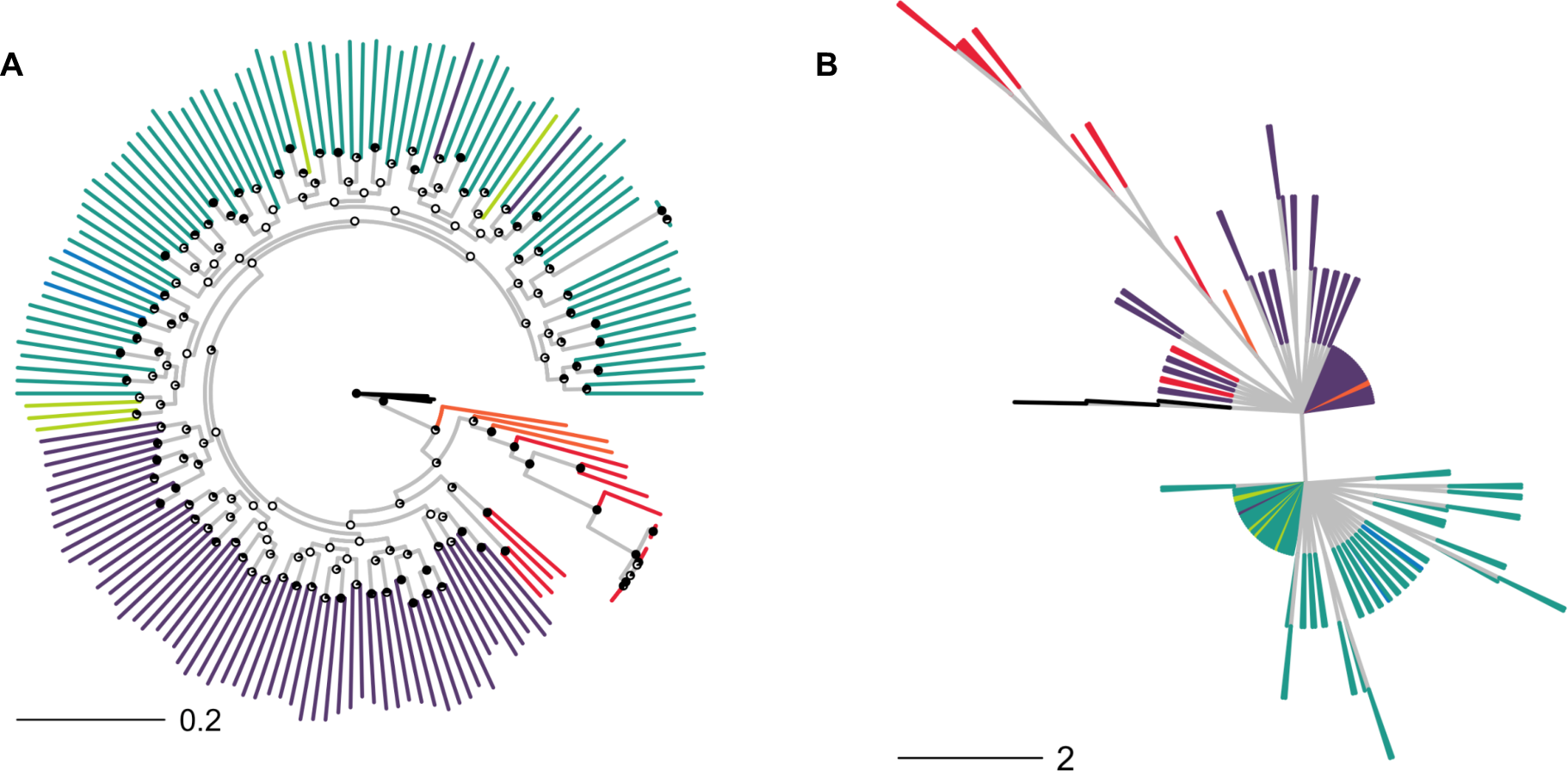

Supplement: S6 Fig — A) RAxML maximum likelihood tree where nodes are pictured with pie charts representing bootstrap values (wherein darker circles hold higher confidence) from 1000 bootstraps. Tree is based on 40,008 high-quality internal SNPs. B) Consensus tree based on 1000 bootstraps. (TIF) [file pntd.0008506.s010.tif]

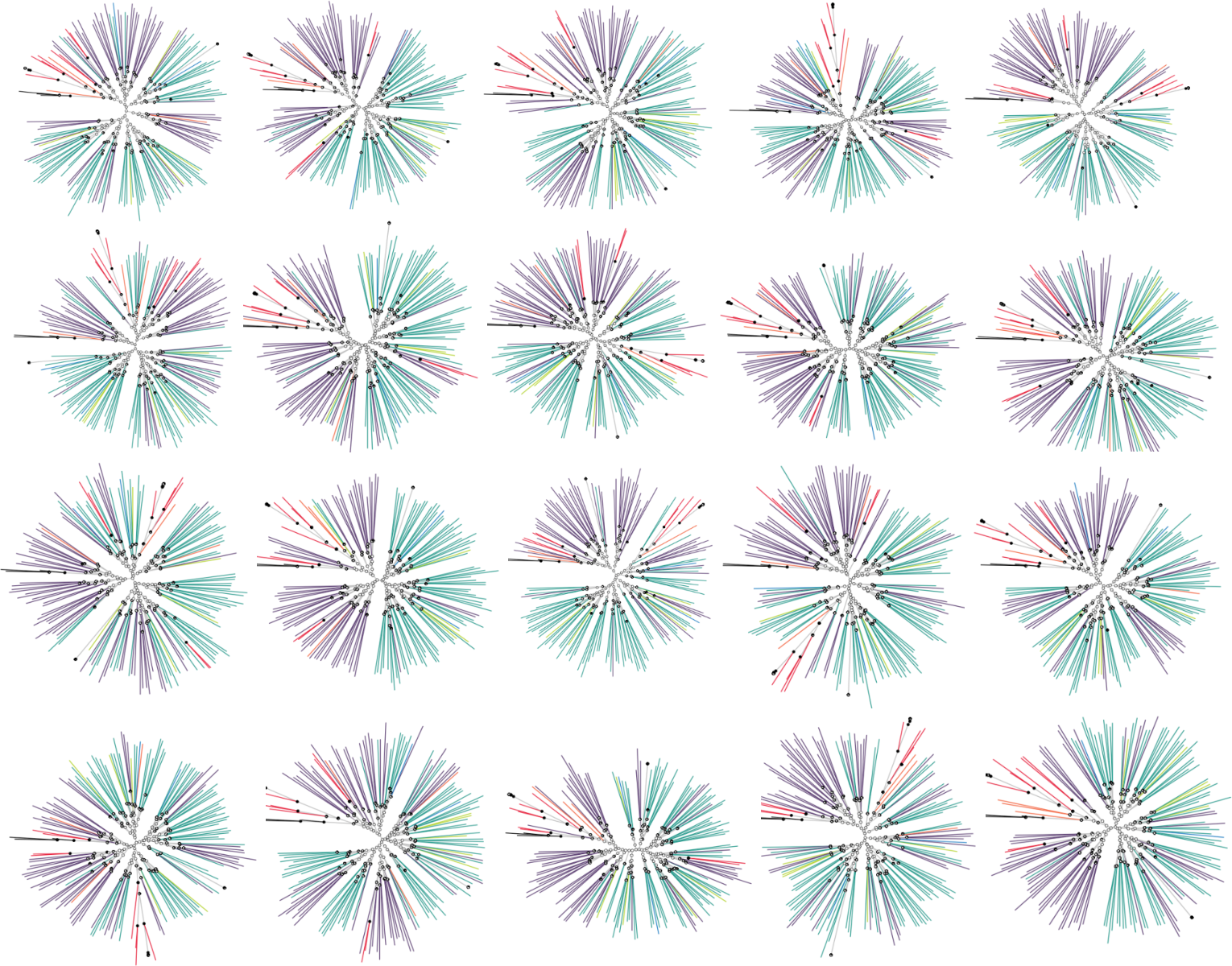

Supplement: S7 Fig — Each tree was constructed with a new set of 5000 random SNPs regardless of MAF. Colors: Salmon (CMB), Orange (China), Lavendar (Thailand), Teal (Cambodia), Blue (Laos), Green (Vietnam). Nodes are pictured with pie charts representing bootstrap values (wherein darker circles hold higher confidence) from 1000 bootstraps. Tree is based on 40,008 high-quality internal SNPs. (TIF) [file pntd.0008506.s011.tif]

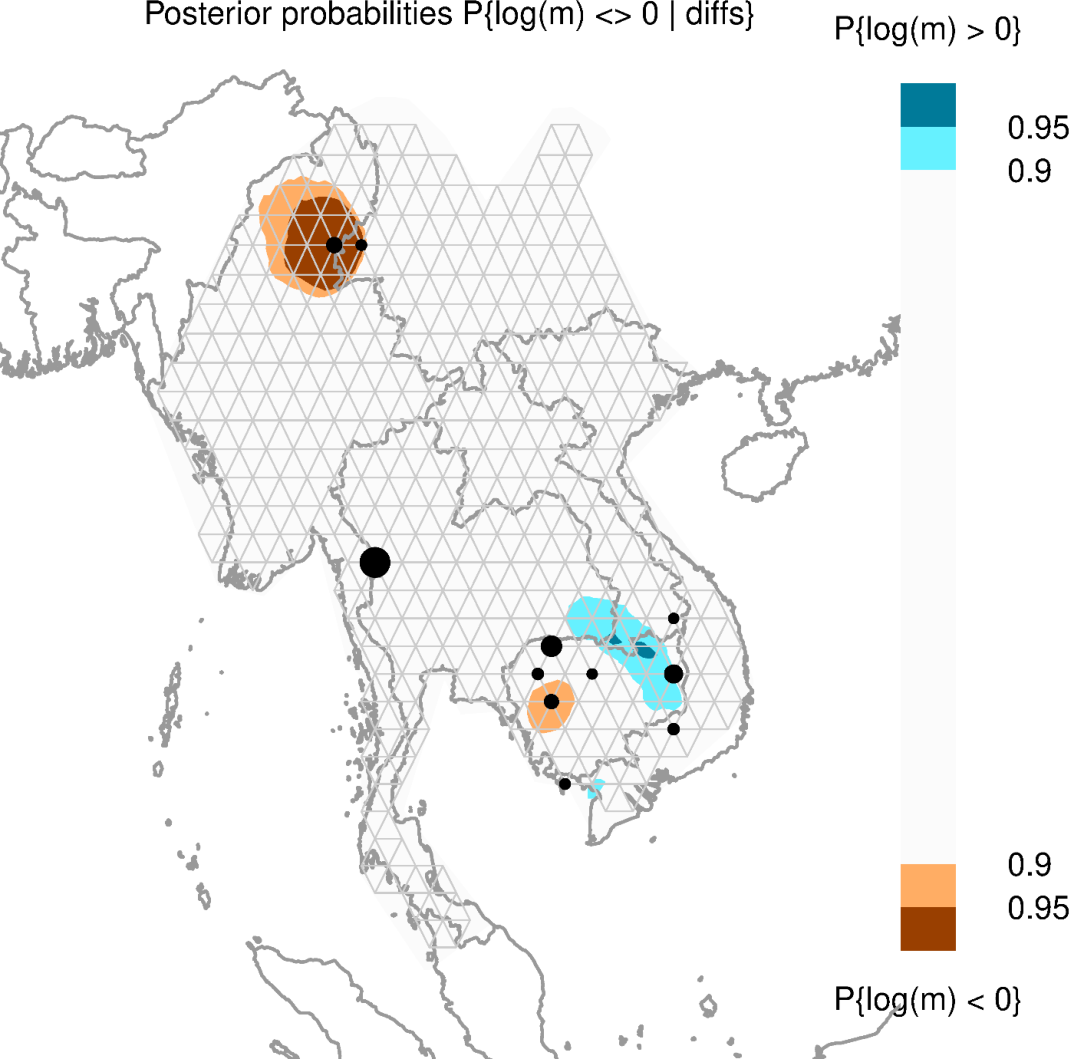

Supplement: S8 Fig — (TIF) [file pntd.0008506.s012.tif]

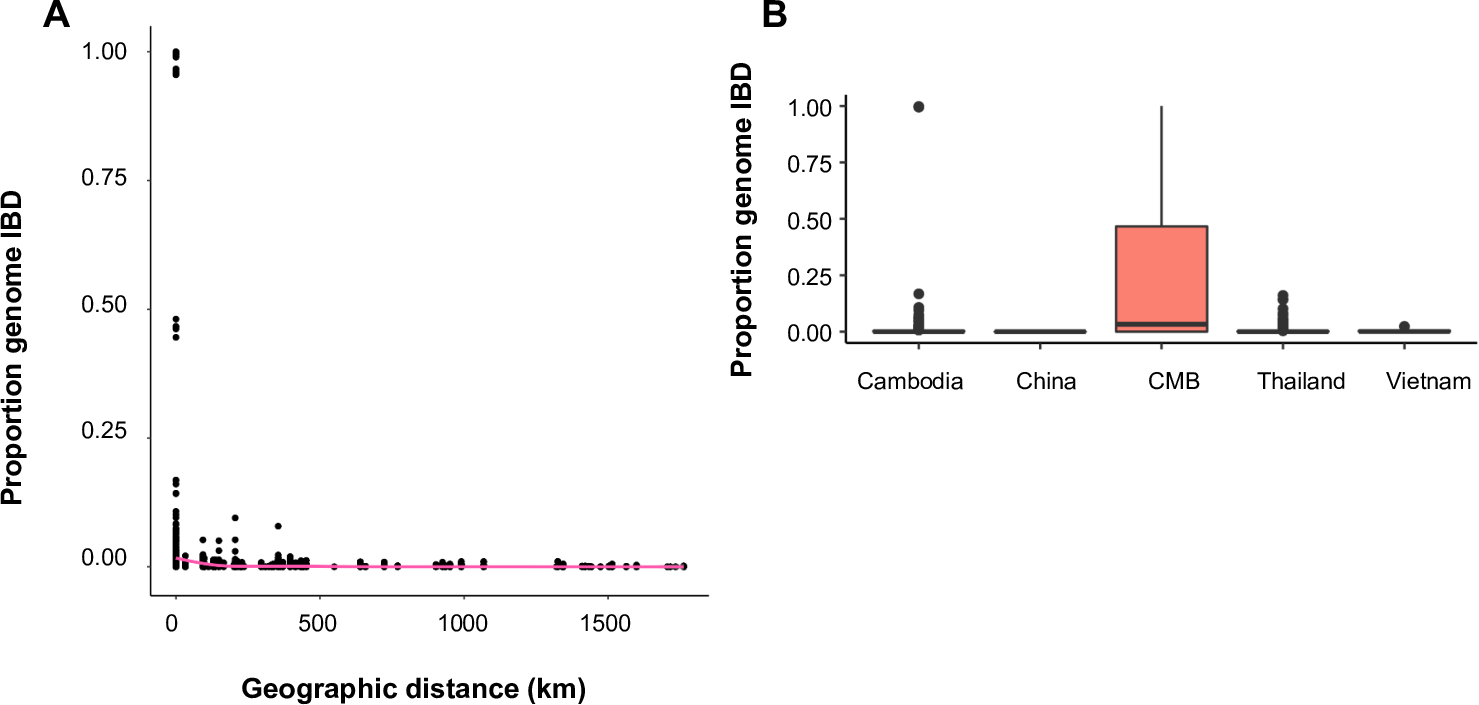

Supplement: S9 Fig — Hmm-IBD estimates were made under the assumption of two populations which we defined by those in the CMB and those not in the CMB as supported by admixture analysis. (A) Geographical distance in kilometers compared to proportion of genome predicted to be IBD. (B) IBD-sharing among parasites from the same sample sites (distance = 0) in each location. (TIF) [file pntd.0008506.s013.tif]

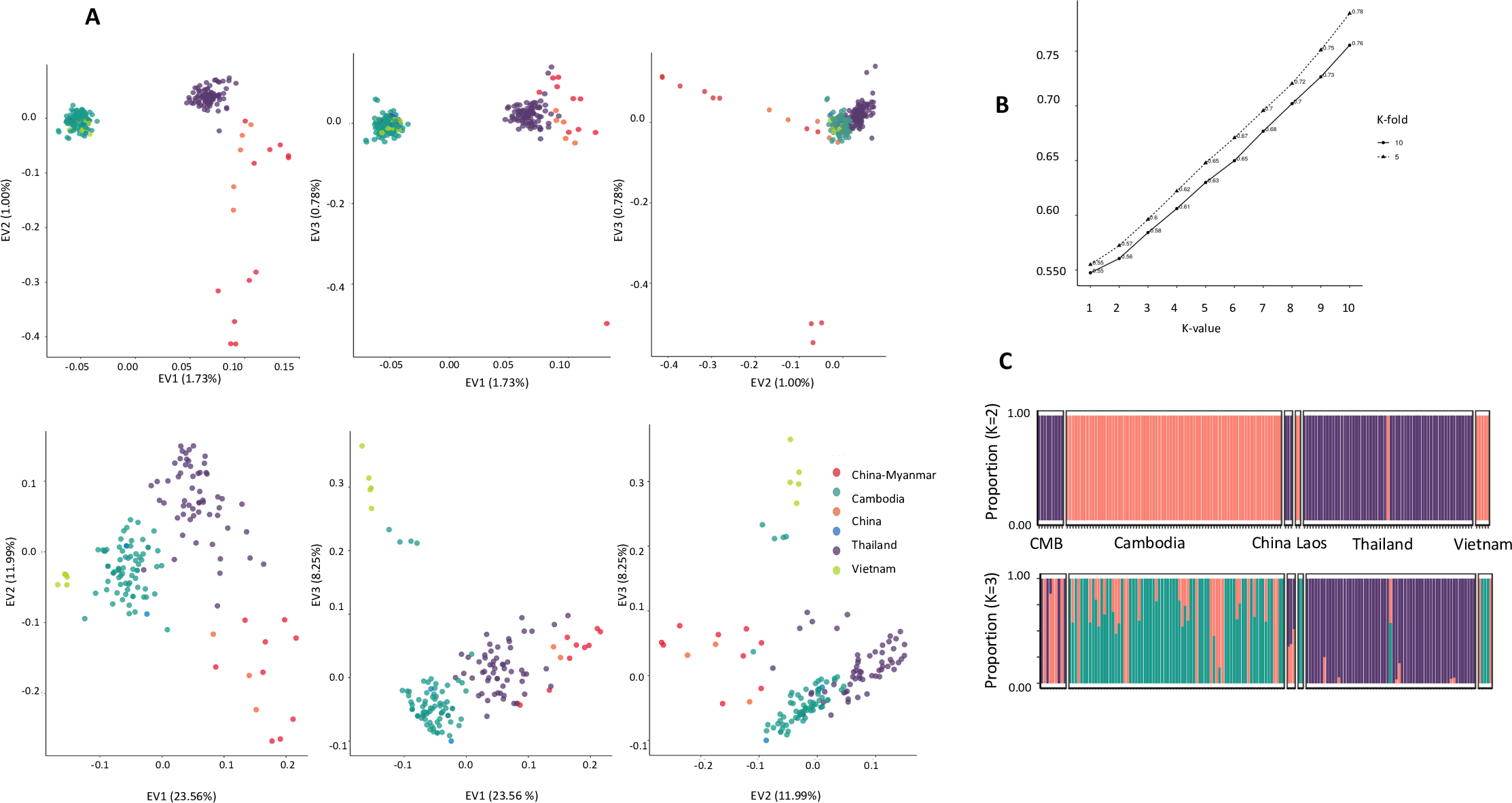

Supplement: S10 Fig — A) Principle component analysis with full SNP set (top) and barcode only (bottom). B) Admixture cross-validation errors at various k-values. C) Admixture analyses at k = 2 and k = 3. (TIF) [file pntd.0008506.s014.tif]

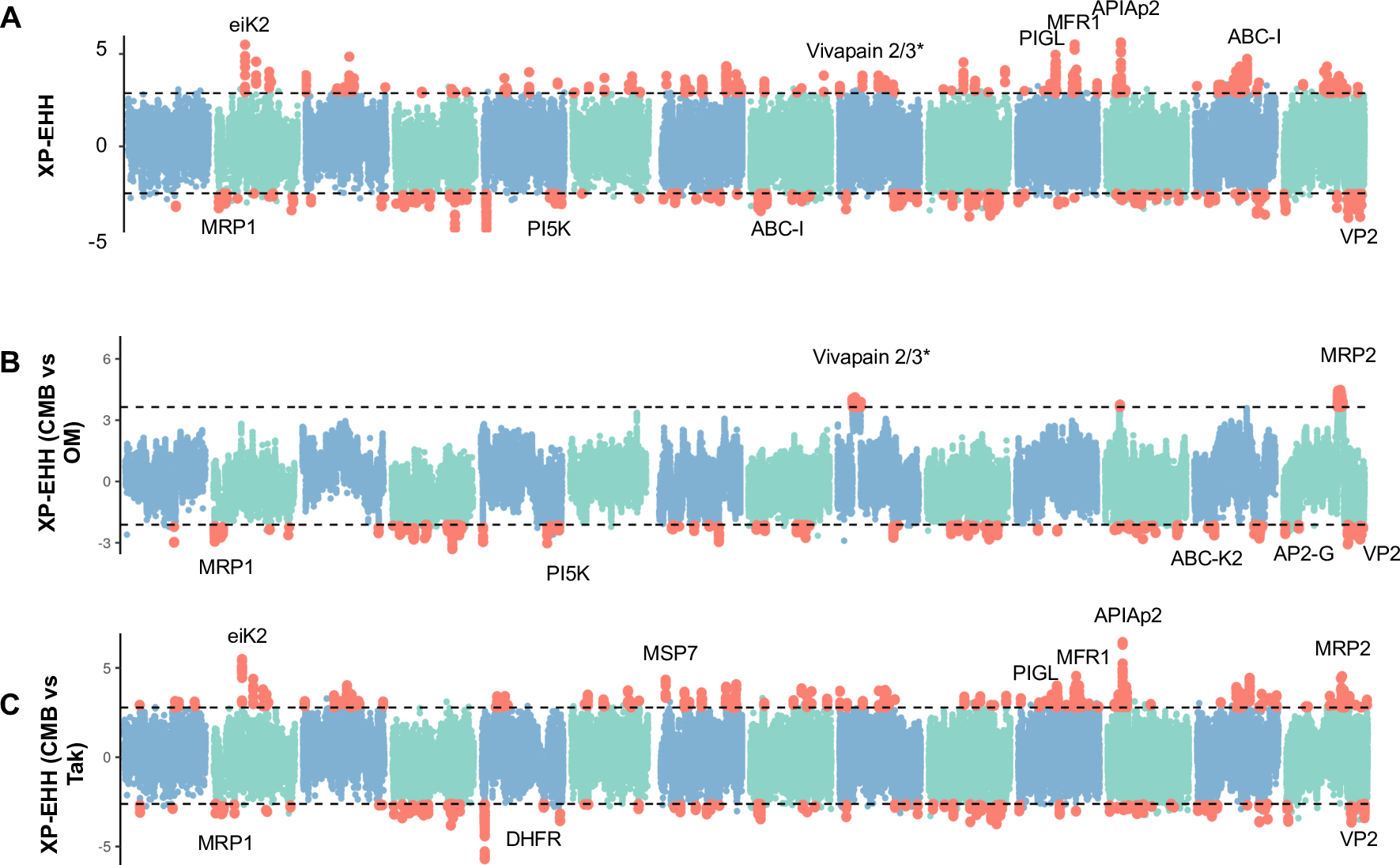

Supplement: S11 Fig — XP-EHH for the China-Myanmar border using A) the rest of the GMS as reference, B) Oddar Meanchey, Cambodia as reference, and C) Tak Province, Thailand as reference. Red points represent pairs of SNPs within either the top or bottom 0.5% of all SNPs for XP-EHH value. (TIF) [file pntd.0008506.s015.tif]
