# Supplementary material for: Population genomics identifies a distinct Plasmodium vivax population on the China-Myanmar border of Southeast Asia
Source: PLoS Negl Trop Dis. 2020 Aug 3;14(8):e0008506. doi: 10.1371/journal.pntd.0008506 (PMC7425983; doi:10.1371/journal.pntd.0008506)
Supplement: Attachment — Submitted filename: Report Review PNTD-D-20-00486_200422.pdf [file pntd.0008506.s018.pdf]

Report Review PNTD-D-20-00486

[April 22, 2020]

Brashear *et al*. present a comprehensive study on the genomic characterization of the *P. vivax* (Pv) population in a specific area of the China-Myanmar border (CMB) in comparison with Pv populations in the Greater Mekong Subregion (GMS) countries Thailand, Cambodia, Vietnam and Laos.

The manuscript is well written: Rationale and aims are clearly outlined in the introduction, methods are all-inclusive and state-of-the-art, and the results are presented in detail and in a logical manner. However, I have some major comments regarding their discussion of their findings and resulting conclusions.

1) The samples have been collected during a Pv outbreak in this region in 2013. Hence, overall results do not come as a surprise to me. Instead of concluding an outbreak from their data, I would have expected to find this information in the 'introduction' or the '’methods' and data be interpreted in view the fact that these are – indeed – samples from a single outbreak. This is, a limitation of the study, particularly in their comparative analyses of the CMB population with the other GSM populations: If infections emerge from a clonal expansion in one population, this can significantly skew further downstream analyses comparing different populations. I suggest to re-analyse the data under different threshold scenarios, with the threshold referring to how independent=unrelated single samples of the CMB population are. Depending on the threshold, (i.e., how many same or highly related samples are removed from the CMB population) your overall population size may shrink, but the remaining samples may be more representative of the CMB population.

2) The authors claim their 36 SNP panel to be an ideal set for this region for monitoring the gene flow across borders. However, for the very same reason (i.e., clonal expansion), this SNP set my not be able to be used in other regions, even not along the entire CMB. I acknowledge that the authors state that the SNP set has to be validated further in the whole of the GMS. However, I'd prefer the authors to be a bit more cautious with this conclusion, particularly in view of the fact that the Thai data set comes from the Thai-Myanmar border area where significant gene flow across borders has been shown.

More minor comments below:

<u>General</u>

3) The authors should write in full an abbreviation the first time it occurs in the manuscript.

4) There seems to be a 'glitch' in the reference managing program.

<u>Methods</u>

5) Data availability: Data are not available in the NCBI database under bioproject PRJNA603279. Will they be uploaded upon publication? I suggest uploading to the European Nucleotide Archive (ENA).

6) Lines 147-148: The first sentence can be deleted. Stated in lines 133-134.

<u>Results</u>

7) Table titles should appear on top of the table and footnotes at the bottom.

8) Authors compared their regional 36 SNP barcode with the Broad Institute global 42 SNP barcode and the LSTMH 72 SNP barcode. It would be interesting to see how it compares to the recently uploaded 65 SNP 9 (full) and 28 SNP (core) barcodes [https://www.biorxiv.org/content/10.1101/776781v1].

9) Line 366: “services” should read “surfaces”.

10) Lines 426 and 435: REF #38, #49, and #40 can be omitted.

11) Line 435: “*pfmdr*1” should read “*pvmdr*1. Please note: The role of *pvmdr*1 in mediating chloroquine resistance in *P. vivax* is still controversial; the authors should consider this.

12) Figure 3C: Data could be visualized better in a rooted tree.

Discussion

See comments #1 and #2.

13) Line 515: Change to “…and used this information to characterize the *P. vivax* population in this region.”

14) Line 557: Ref #36, #43, and #44 should be removed.

15) Line 609: Change to “…emerging chloroquine resistance in the northeaster Myanmar *P. vivax* population”.
