# Supplementary material for: Population genomics identifies a distinct Plasmodium vivax population on the China-Myanmar border of Southeast Asia
Source: PLoS Negl Trop Dis. 2020 Aug 3;14(8):e0008506. doi: 10.1371/journal.pntd.0008506 (PMC7425983; doi:10.1371/journal.pntd.0008506)
Supplement: Attachment — Submitted filename: Response to Reviewers.pdf [file pntd.0008506.s019.pdf]

Responses to Reviewers' Comments

We want to thank the three reviewers for their support and constructive comments!

Reviewer #1:
This manuscript describes 21 new genome-wide sequences of Plasmodium vivax isolates from the China-Myanmar border (CMB) in Asia. Newly generated genome data are compared with other ,publicly available sequences from Cambodia (n = 136, Thailand (n = 91), Vietnam (n = 10), Yunnan Province of China (n = 5), and Laos (n = 2) to provide a comprehensive overview of the population genomics of this species in the Greater Mekong Region (GMR). Methods used for generating and analyzing genome data are entirely appropriate and up to date. Results are clearly presented and show relatively little gene flow between CMB parasites and those from neighboring areas in GMR. Interestingly, instances of clonal propagation of CMB parasites have been observed. This is a well-done study submitted by an experienced team of population geneticists. They have generated a limited number of new genome-wide sequences of Plasmodium vivax from an understudied population (CMB), but extensively compare their findings with those from other sites in the GMR. The overall picture they reveal is quite interesting, with relatively little gene flow between the CMB populations and the other P. vivax populations in the region.

1. Discussion is comprehensive and appropriate. I would like to see a little more about the epidemiological implications of these findings.
Answer: Thank you for this comment. We have added more discussion on the relevance of the findings for malaria elimination from this region.

Reviewer #2:
Brashear et al. present a comprehensive study on the genomic characterization of the P. vivax (Pv) population in a specific area of the China-Myanmar border (CMB) in comparison with Pv populations in the Greater Mekong Subregion (GMS) countries Thailand, Cambodia, Vietnam and Laos. The manuscript is well written: Rationale and aims are clearly outlined in the introduction, methods are all-inclusive and state-of-the-art, and the results are presented in detail and in a logical manner. However, I have some major comments regarding their discussion of their findings and resulting conclusions.

1. The samples have been collected during a Pv outbreak in this region in 2013. Hence, overall results do not come as a surprise to me. Instead of concluding an outbreak from their data, I would have expected to find this information in the 'introduction' or the ''methods' and data be interpreted in view the fact that these are – indeed – samples from a single outbreak. This is, a limitation of the study, particularly in their comparative analyses of the CMB population with the other GSM populations: If infections emerge from a clonal expansion in one population, this can significantly skew further downstream analyses comparing different populations. I suggest to re-analyse the data under different threshold scenarios, with the threshold referring to how independent=unrelated single samples of the CMB population are. Depending on the threshold, (i.e., how many same or highly related samples are removed from the CMB population) your overall population size may shrink, but the remaining samples may be more representative of the CMB population.
Answer: Thank you for this comment. We agree that samples that are potentially resulted from expansion of some clones can influence various conclusions, particularly regarding population structure. In accordance, we have included a supplemental figure detailing the outcome of Fst and Population analyses wherein we selected a sample from the CMB to represent clones from potential expansion (S10 figure and S3 Table). Ultimately, while reducing the IBD clusters did reduce the complexity of structure, particularly in the admixture analysis, the presence of separation between the CMB, Thailand and Cambodia is still readily apparent. Additionally, Fst values, while lower than they are with IBD cluster control, are still higher between the CMB and all other regions than any of the other locations compared to one another.

We do also think this is an important point, and have expanded our sections in both introduction and discussion.

2. The authors claim their 36 SNP panel to be an ideal set for this region for monitoring the gene flow across

borders. However, for the very same reason (i.e., clonal expansion), this SNP set my not be able to be used in other regions, even not along the entire CMB. I acknowledge that the authors state that the SNP set has to be validated further in the whole of the GMS. However, I'd prefer the authors to be a bit more cautious with this conclusion, particularly in view of the fact that the Thai data set comes from the Thai-Myanmar border area where significant gene flow across borders has been shown.
Answer: We completely agree with your comment. We recognize that the 42 SNP barcode can well separate most of the world Pv populations, we sought to identify a SNP set that is useful in the GMS to help distinguish the populations to help with the regional malaria elimination. Even though we used LD pruning, which should mitigate some effects of clonal expansion on the overall barcode efficacy, we recognize this is still a potential limitation to our barcode. Therefore, we have included in our supplemental figure a test of the 36 SNP barcode with IBD clusters collapsed into representatives to confirm it is still effective at distinguishing between populations (S10 Figure A). Our results suggest that populations are still distinguishable using this barcode despite the removal of IBD clusters. This also testifies that there is a strong gene flow in the GMS (also revealed from our recent population study using microsatellite markers), partially due to the large-scale migration in the whole GMS. We have thus discussed this topic in more detail.

3. The authors should write in full an abbreviation the first time it occurs in the manuscript. There seems to be a 'glitch' in the reference managing program.
Answer: We went through the revision carefully to fix these issues. The reference "glitch" might be due to use of different Endnote libraries by the co-authors. We made a final check to make sure that references are correctly cited.

4. Data availability: Data are not available in the NCBI database under bioproject PRJNA603279. Will they be uploaded upon publication? I suggest uploading to the European Nucleotide Archive (ENA).
Answer: These data have been uploaded and will be made available upon editorial acceptance.

5. Lines 147-148: The first sentence can be deleted. Stated in lines 133-134.
Answer: This has been fixed, thank you.

6. Table titles should appear on top of the table and footnotes at the bottom.
Answer: Corrected.

7. Authors compared their regional 36 SNP barcode with the Broad Institute global 42 SNP barcode and the LSTMH 72 SNP barcode. It would be interesting to see how it compares to the recently uploaded 65 SNP 9 (full) and 28 SNP (core) barcodes
Answer: Thank you for this recommendation. The 42 SNP Broad barcode only had 9 callable SNPs which were largely monomorphic and were thus not further analyzed. We additionally analyzed the 28 SNP barcode as well as the 51 SNP barcode. Of those, about 1/3 of locations from either barcode were not callable within out dataset. Preliminary PCA analyses demonstrated very little separation between GMS countries. We included the SNP presence in our results and a discussion of this barcode in the discussion.

8. Line 366: "services" should read "surfaces".
Answer: Corrected.

9. Lines 426 and 435: REF #38, #49, and #40 can be omitted.
Answer: Thank you, we have removed the superfluous references.

10. Line 435: "pfmdr1" should read "pvmdr1. Please note: The role of pvmdr1 in mediating chloroquine resistance in P. vivax is still controversial; the authors should consider this.
Answer: we have fixed this typo. We have also noted this controversy in our discussion.

11. Figure 3C: Data could be visualized better in a rooted tree.
Answer: Thank you, we have rooted our tree on Malagasy samples in order to make relationships more clear.

12. Line 515: Change to "…and used this information to characterize the P. vivax population in this region."
Answer: We have modified this sentence.

13. Line 557: Ref #36, #43, and #44 should be removed.
Answer: yes, removed.

14. Line 609: Change to "…emerging chloroquine resistance in the northeaster Myanmar P. vivax population".
Answer: yes, changed.

Reviewer #3:
This study examined the genetic relatedness and genomic features of P. vivax including 21 new genomes from the CMB and 200+ published genomes from other parts of the Great Mekong Subregion. The authors found clear differentiation between the CMB and eastern GMS samples. In particularly, they identified a panel of 36 high-performance SNPs that can provide country-level resolution. They examined further selection genome-wise and identified a number of resistance genes that have undergone different level of fixation among regions. I applaud the authors doing a thorough analyses on the vivax genomes in the region. The manuscript is very well written and easy to follow. Results corroborate some of the previous findings. I have a few minor comments and suggestions.

1. L188-189 What is the reason of including polyclonal samples? Whether Fst and PCA show similar results if only monoclonal samples are included.
Answer: The primary concern for polyclonal samples is that a variant may be called representing primarily one candidate in one part of the genome, but would be called representing primarily another in a separate part of the genome. Since Plink (and vcftools) calculates Fst for each SNP independently of one another before averaging SNPs together this should not affect Fst results. Meanwhile, the especially small population sizes of Vietnam and, in the case of PCA, China, would reduce our confidence in the results. We additionally kept the polyclonal samples for PCA in order to main consistency with Fst results, and it is worth noting that previous papers (including the Pearson 2016 and Hupalo 2016 papers) have kept all samples regardless of clonality to maximize sample size. That being said, we realize that polyclonal samples could cause chimeric individuals and alter PCA results. We have therefore added a supplemental figure (S3 Figure) which replicates PCA cluster topology using only monoclonal samples.

2. L288-290 It's not clear why compare Fws values between GMS countries? Fws values are purposed to identify mono vs. polyclonal samples. The comparison of polyclonal rate makes sense among regions, but it's not meaningful to compare Fws.
Answer: Thank you for this comment. Coinfection with two unrelated clones will frequently result in a lower Fws than coninfection with two related clones because more SNPs will be divergent between the two. This is a concept initially explored by Manske et al. (2012, Nature) and demonstrated effectively by Pearson et al. (2016, Nature Genetics). The high distribution of Fws values among samples in the CMB, even those with an Fws below the threshold for monoclonality, could be related due to clonal expansion in a population resulting in lower parasite diversity. We have expanded our discussion of clonality and Fws in order to accommodate this understanding.

3. I suggest insert a Table that show complete information (including position, gene function, mutant frequency in each of the five countries/regions) of the 36 high-performance SNPs.
Answer: Thank you for this great point. We have converted what was initially S3 Table (chromosomes and positions) into a secondary sheet in S2 File which now contains allele frequency data and SNPeff predictions in addition to positions for each of the 53 SNPs (with the 36 highlighted).

4. Figure 1 – Maps need better resolution and site labels to navigate the readers. Likewise, for other figures that need higher quality

Answer: Thank you for this comment. We used more detailed maps for the GMS data and included 2016 incidence data in order to give more context to sample locations.

5. L526-527 Any information on the malaria history that the CMB cases are relapse and a possible correlation between monoclonality and relapse? Along with this point, in L561 - any information on possible relapse cases among the samples or references that support the statement that relapses might reduce the correlation between geographic distance and IDB.
Answer: Unfortunately, we do not have metadata that would make us confident stating that the clonally expanded parasite is from relapse or from a new infection. Neafsey et al. 2012 (Nature genetics) described a higher diversity within *P. vivax* than *P. falciparum* and attributed it to two main causes, 1) less population bottlenecks (i.e. drug pressure) and 2) relapse allowing the reintroduction of alleles. We have expanded on this in our discussion.

6. L539-544 While the tree shows monophyly of CMB and Yunnan samples, both K2 and K3 admixture plots indicate distinct clusters for the two regions. Need to provide explanation.
Answer: Thank you for this comment. There are few artefacts of the two methods/results that account for this discrepancy. First, while the maximum likelihood tree suggested monophyly, the bootstrap support was not high, and in our consensus tree constructed from all nodes with above 50% bootstrap support, this was not distinguishable, suggesting the China samples have a genetic profile intermediary to the CMB and Thailand samples. This is also supported by higher k-value admixture plots (6+) wherein the China sample takes on a separate population that has some presence in both the Thailand and CMB samples. The overall low sample number for the China samples also lends itself to be less powerful within the construction of admixture populations. We have added a discussion of this in our discussion section.

7. L143 21 or 23 new samples?
Answer: 23 new samples, 21 with viable whole genome coverage. We have clarified this.
