# Supplementary material for: Population genomics identifies a distinct Plasmodium vivax population on the China-Myanmar border of Southeast Asia
Source: PLoS Negl Trop Dis. 2020 Aug 3;14(8):e0008506. doi: 10.1371/journal.pntd.0008506 (PMC7425983; doi:10.1371/journal.pntd.0008506)
Supplement: Attachment — Submitted filename: Response to Reviewers.docx [file pntd.0008506.s020.docx]

**Responses to Reviewers’ Comments**

We want to thank reviewers for their support and constructive comments!

**Reviewer #2:**

Methods are all-inclusive, appropriate, and state-of-the-art.
Results are presented clearly in great detail and in a logical manner.

Thank you for these comments.

The discussion is appropriate and all-inclusive. However, I miss the discussion of the limitations of their study. See 'general comments' below.

The authors adequately addressed all comments of the three Reviewers and modified the manuscript accordingly. Apart from minor editorial changes that are required (i.e., typos, etc.), there a couple of additional comments I would like to add:

The authors compare their 36-NP panel/barcode with the global
a) 42-SNP barcode (Neafsey et al., 2012),
b) 71-SNP barcode (Diez Benavente et al., 2020), and
c) 65-SNP (full) and 28-SNP (core) barcodes (Trimarsanto et al., bioRxiv 2020).

1) Apparently, not all SNPs of the alternative barcodes were callable.
What was the reason for this?

Thank you for noting this. The lack of polymorphisms called is likely reflective of parasites which largely have no variation at that locus. The visualization of SNP allele frequencies in Benavente et al. 2020 has an intuitive representation of differing allele frequencies, in which even many of their Southeast Asian samples were completely fixed within Southeast Asia. It’s also important to note that we filtered out SNPs which had a minor allele frequency under 0.01 as part of our quality control in order to reduce erroneous calls, so even if 0.9% of samples had an imported allele at these locations, they would be filtered out. We modified our language in order to reflect this possibility.

2) They claim that the Matthew correlation coefficient (MCC) scores were lower in these alternative barcodes than in their 36-SNP barcode. However, there is no mention in the 'Methods" about how they calculated the MCC scores and more importantly, they do not give the MCC score of their 36-SNP barcode.

Thank you for bringing this to our attention. This is a misunderstanding and we have reworded this section to explain. At no point did we ourselves use machine learning to create or evaluate our barcode, and therefore the MCC metric was never used in our methods. Our brief comparison of barcodes was done by enumerating callable SNPs and informal visualization. Additionally, in some cases the authors of the barcodes note that Southeast Asian countries are poorly resolved which necessitates a locally-derived barcode. For example, we referenced MCC because the Trimarsanto paper itself found that their barcode performed most poorly for Cambodia and Vietnam out of all of the countries tested, and it had very small sample sizes for China and the CMB. In order to quantify this they used MCC, a metric which measures the agreeance between a machine learning model’s expected and produced result. They found that while the median performance MCC was 0.944 for the 28 SNP barcode and 0.979 for the 51 SNP barcode, Vietnam and Cambodia performed below 0.9 and these results are even worse for the broad institute barcode.
